# Suppression of neuropeptide production by quercetin in allergic rhinitis model rats

**DOI:** 10.1186/s12906-016-1123-z

**Published:** 2016-05-20

**Authors:** Misako Kashiwabara, Kazuhito Asano, Tomomi Mizuyoshi, Hitome Kobayashi

**Affiliations:** Graduate School of Nursing and Rehabilitation Sciences, Showa University Graduate School, Midori-ku, Yokohama, 226-8555 Japan; Department of Otolaryngology, School of Medicine, Showa University, Shinagawa-ku, Tokyo, 142-8555 Japan; Division of Physiology, School of Nursing and Rehabilitation Sciences, Showa University, 1865 Touka-Ichiba, Midori-Ku, Yokohama, 226-8555 Japan

**Keywords:** Quercetin, Substance P, Calcitonin gene-related peptide, Nerve growth factor, Nasal allergy-like symptom, Suppression, Rat

## Abstract

**Background:**

Quercetin, a dietary flavonoid found in many fruits, red wine and onion, among others, has been reported to have potent anti-oxidant, anti-viral and anti-cancer effects. Although quercetin is also reported to have anti-inflammatory and anti-allergic effects, the precise mechanisms by which quercetin favorably modify the clinical conditions of allergic diseases such as allergic rhinitis (AR). The present study was designed to examine the influence of quercetin on the development of AR by using AR model rats.

**Methods:**

Sprague-Dawley (SD) rats were sensitized with toluene 2,4-diisocyanate (TDI) by intranasal instillation of a 10 % TDI in ethyl acetate in a volume of 5 μl once a day for 5 consecutive days. This sensitization procedure was repeated after a 2-day interval. After 5 days of the second sensitization, rats were treated with various doses of quercetin once a day for 2 to 7 days. Nasal allergy-like symptoms, which were induced by bilateral application of 5 μl of 10 % TDI in ethyl acetate, were assessed by counting sneezing and nasal rubbing behaviors for 10 min just after TDI nasal challenge. The levels of substance P (SP), calcitonin gene-related peptide (CGRP) and nerve growth factor (NGF) in nasal lavage fluids obtained 6 h after TDI nasal challenge was examined by ELISA.

**Results:**

Oral administration of quercetin for 5 and 7 days, but not 2 and 3 days, could inhibit sneezing and nasal rubbing movements, which were increased by TDI nasal challenge. The minimum dose that caused significant inhibition was 25 mg/kg. Oral administration of quercetin at more than 25 mg/kg for 5 days significantly inhibited the increase in SP, CGRP and NGF contents in nasal lavage fluids induced by TDI nasal challenge.

**Conclusion:**

The present results strongly suggested that quercetin will be a good candidate for the supplement on the management and treatment of allergic diseases, especially AR.

## Background

Allergic rhinitis (AR) is accepted to be an IgE-mediated inflammatory disease in the nasal mucosa and characterized by intense infiltration and activation of inflammatory cells such as mast cells, and eosinophils, among others [1]. Although AR is not life-threatening disease, it can deteriorate the quality of life and an economic burden through the clinical symptoms such as sneezing, rhinorrhea, itching and nasal congestion [1, 2]. These clinical conditions are well known to be primarily induced by chemical mediators such as histamine and leukotrienes, which are secreted by mast cells, basophils and eosinophils [1]. In addition to the secretion of chemical mediators, these cells also release several types of cytokines and chemokines, which are responsible for the amplification and the persistence of allergic inflammatory responses in nasal mucosa [1]. On the other hand, there is much evidence that nasal mucosa is innervated by sensory, sympathetic and parasympathetic nerves. After stimulation with aeroallergen, sensory nerves transmit signals generating sensations such as itching and motor reflexes, including sneezing [3–6]. Antigenic stimulation on sensory nerves also causes axonal reflex to produce neuropeptides, substance P (SP) and calcitonin gene-related peptides (CGRP), which are responsible for vasodilation, edema and activation of inflammatory cells in nasal mucosa [3–6].

Quercetin is one of the well-characterized flavonoids and found in onion, red wine and mulberry, among others [7]. It is reported that quercetin exerts many beneficial activities on human health, including anti-oxidative, anti-diabetic and anti-cancer activities [8–10]. In regard to allergic diseases, quercetin is reported to attenuate clinical conditions of allergic diseases such as AR through its suppressive effects on the release of inflammatory cytokines and chemical mediators from mast cells and eosinophils after immunological stimulation [11, 12]. Therapeutic potential of quercetin on allergic airway diseases is also observed in animal experimental models of asthma, in which oral administration of quercetin showed the inhibitory action on bronchial hyper-reactivity to specific allergen [13, 14]. It is also reported that quercetin could effectively block the development of anaphylactic responses against peanuts in the experimental mouse model and in vitro cell lines [15, 16]. Although these reports strongly suggest that quercetin will be a good candidate as a potential drug to allergic diseases, the influence of quercetin on neuropeptide productions is poorly understood. In the present study, therefore, we examined the influence of quercetin on neuropeptide production by using AR model rat.

## Methods

### Animals

Specific pathogen free, male Sprague-Dawley (SD) rats, 5 weeks of age were purchased from CLEA JAPAN Co., Ltd. (Tokyo, Japan). These animals were maintained in our animal facilities at 25 **±** 2 °C with 55 **±** 10 % humidity under a 12-h light/dark cycle and were allowed free access to tap water and standard laboratory rodent chow (Oriental Yeast Co., Ltd., Tokyo, Japan) throughout the experiments. Each control and each experimental group consisted of five rats. All animal experiments were approved by the Ethics Committee for Animal Experiments of Showa University (Approved No. 05112).

### Sensitization and challenge procedures

SD rats were sensitized with toluene 2,4-diisocyanate (TDI) according to the method described previously [17]. Briefly, 5 μl of a 10 % TDI (Wako Pure Chemicals Co., Ltd., Osaka, Japan) solution in ethyl acetate (Wako Pure Chemicals Co., Ltd.) was instilled bilaterally into the nasal vestibule once a day for 5 consecutive days. This sensitization procedure was repeated after a 2-day interval. To induce nasal allergy-like symptoms, 5 μl of 10 % TDI solution in ethyl acetate was applied bilaterally on the nasal vestibule of sensitized rats. Control rats were treated with ethyl acetate only by the same procedure.

### Treatment of rats with agents

Quercetin purchased from Sigma-Aldrich Co., Ltd. (St. Louis, MO, USA) was well mixed with 5 % tragacanth gum solution at a concentration of 7.5 mg/ml. Rats were orally administered with either 10, 20, 25 or 30 mg/kg of quercetin once a day for 2 to 7 days via a stomach tube in a volume not exceeding 1.0 ml. Olopatadine hydrochloride (OH), a second generation histamine H_1_ receptor antagonist, was used for treatment as a positive control. OH for human use was purchased from Kyowa Hakko Kirin Co., Ltd. (Tokyo, Japan). This was dissolved in distilled water at a concentration of 10 mg/ml and administered orally into rats at a dose of 10 mg/kg once a day for 5 days via a stomach tube [19]. Treatment was started 5 days after the final second sensitization with TDI.

### Assay for nasal symptoms

Nasal allergy-like symptoms were assessed by counting numbers of sneezing and nasal rubbing movements for 10 min just after TDI nasal challenge. The experimental rats were placed into the plastic animal cage (35 × 20 × 30 cm) for about 10 min for acclimation. After the nasal instillation of 10 % TDI solution in ethyl acetate in a volume of 5 μl, rats were placed into the plastic cage (one animal/cage) and the number of sneezes and nasal rubbing movements for 10 min were counted [19, 20].

### Preparation of nasal lavage fluids

The rats were killed by intraperitoneal injection with 100 mg/kg sodium pentobarbital (Kyoritsu Seiyaku Co., Ltd., Tokyo, Japan) 6 h after TDI nasal challenge. The trachea was exposed and cannulated to introduce 1.0 ml phosphate buffered saline. The lavage fluid exiting the nares was collected and centrifuged at 3000 rpm for 15 min at 4 °C. After measuring IgA by ELISA (Bethyl Lab., Inc., Montgomery, TX, USA), the fluids were stored at -40 °C until used.

### Assay for neuropeptides

Substance P (SP), calcitonin gene-related peptide (CGRP), and nerve growth factor (NGF) in nasal lavage fluids were examined by commercially available ELISA test kits according to the manufacturer's recommendations. SP ELISA test kits were obtained from ENZO Life Science Inc. (Farmingdale, NY, USA), CGRP from Phoenix Pharmaceuticals, Inc. (Burlingame, CA, USA) and NGF from Chemicon International Inc. (Temecula, CA, USA), and the minimum detectable levels of these ELISA test kits was 8.04 pg/ml for SP, 0.01 ng/ml for CGRP and 10.0 pg/ml for NGF.

### Statistical analysis

The results are shown as the mean **±** standard errors of the means (SEM) of five rats/group. Statistical analyses were performed with analysis of variance (ANOVA) followed by Bonferroni correction. A value of *P* < 0.05 was considered statistically significant.

## Results

### Influence of quercetin on the development of TDI-induced nasal allergy-like symptoms

**Fig. 1:**
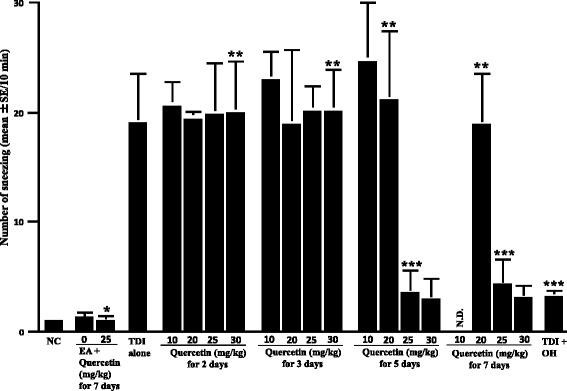
Influence of quercetin on the increase in sneezing after toluene 2,4-diisocyanate (TDI) nasal instillation in sensitized rats. TDI-sensitized rats were treated with various doses of quercetin for 2 to 7 days before TDI provocation. Olopatadine hydrochloride (OH) was administered orally into TDI-sensitized rats once a day for 5 days at a single dose of 10 mg/kg before the provocation. The number of sneezing was counted for 10 min just after TDI nasal challenge. The data are expressed as the mean **±** standard errors of the means of five rats/group. NC: non-sensitized, non-treated control; EA: ethyl acetate; OH: olopatadine hydrochloride; N.D.: not done; *: not significant (P > 0.05) as compared with EA alone, not treated control; **: not significant (P > 0.05) as compared with TDI alone; ***: significant (P < 0.05) as compared with TDI alone

**Fig. 2:**
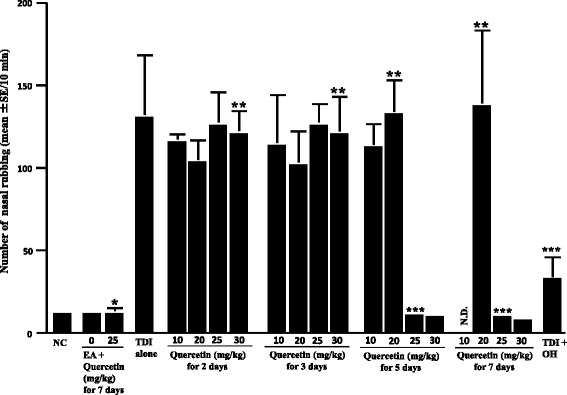
Influence of quercetin on the increase in nasal rubbing by toluene 2,4-diisocyanate (TDI) nasal instillation in sensitized rats. TDI-sensitized rats were treated with various doses of quercetin for 2 to 7 days before TDI provocation. Olopatadine hydrochloride (OH) was administered orally into TDI-sensitized rats once a day for 5 days at a single dose of 10 mg/kg before the provocation. The number of nasal rubbing was counted for 10 min just after TDI nasal challenge. The data are expressed as the mean **±** standard errors of the means of five rats/group. NC: non-sensitized, non-treated control; EA: ethyl acetate; OH: olopatadine hydrochloride; N.D.: not done; *: not significant (P > 0.05) as compared with EA alone, not treated control; **: not significant (P > 0.05) as compared with TDI alone; ***: significant (P < 0.05) as compared with TDI alone

The first set of experiments was undertaken to examine whether oral administration of quercetin into TDI-sensitized rats could inhibit the development of nasal allergy-like symptoms, which was induced by nasal antigenic challenge. Nasal symptoms were assessed by counting the numbers of sneezes and nasal rubbing movements for 10 min just after TDI nasal challenge. As shown in Fig. 1, treatment of TDI-sensitized rats with quercetin for 2 and 3 days could not suppress the development of sneezing induced by TDI provocation, even when 30 mg/kg/day of quercetin was used for treatment. However, oral administration of quercetin at more than 25 mg/kg, but not 10 mg/kg and 20 mg/kg, for 5 and 7 consecutive days inhibited the development of sneezing: the numbers of sneezing observed in rats treated with 10 and 20 mg/kg quercetin are nearly identical (not significant) to that in TDI-sensitized, not treated rats but the sneezing observed in TDI-sensitized rats treated with quercetin at 25 mg/kg and more is significantly lower than control rats (Fig. 1). The data in Fig. 1 also showed the suppressive activity of OH, a histamine-H_1_ receptor antagonist, which is used as a positive control, on the development of sneezing. We then examined the influence of quercetin on nasal rubbing movements induced by TDI nasal challenge. As shown in Fig. 2, oral administration of quercetin at more than 25 mg/kg for 5 and 7 consecutive days, but not 2 and 3 days, significantly suppressed the development of nasal rubbing movements and the number of movements were much lower than that observed in TDI-sensitized, non-treated rats (*P* < 0.05). The data in Fig. 2 also showed the attenuating effect of OH on the development of nasal rubbing movements induced by TDI provocation: the number of movements in TDI-sensitized rats treated with olopatadine was significantly lower than control rats (*P* < 0.05).

### Influence of quercetin on neuropeptide appearance in nasal lavage fluids obtained from TDI-sensitized rats

**Fig. 3:**
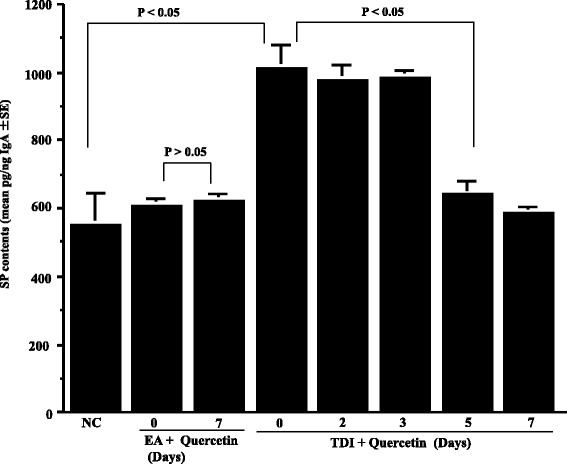
Influence of quercetin on the appearance of substance P (SP) in nasal lavage fluids after toluene 2,4-diisocyanate (TDI) nasal instillation in sensitized rats. TDI-sensitized rats were treated with 25 mg/kg quercetin for 2 to 7 days before TDI provocation. Olopatadine hydrochloride (OH) was administered orally into TDI-sensitized rats once a day for 5 days at a single dose of 10 mg/kg before the provocation. The levels of SP in nasal lavage fluids obtained from sensitized rats 6 h after TDI nasal challenge were measured by ELISA. The data are expressed as the mean pg/ng IgA **±** standard errors of the means of five rats/group. NC: non-sensitized, non-treated control; EA: ethyl acetate

**Fig. 4:**
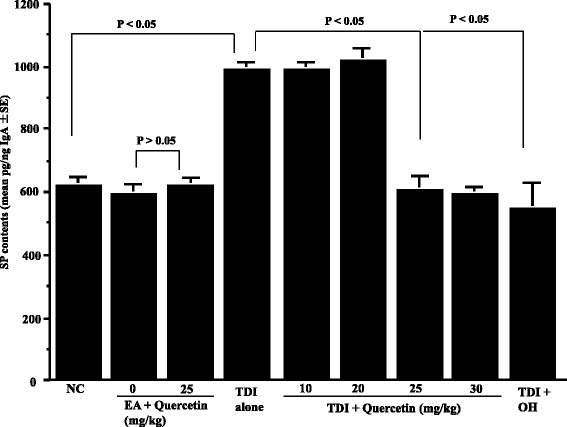
Influence of quercetin on the appearance of substance P (SP) in nasal lavage fluids after toluene 2,4-diisocyanate (TDI) nasal instillation in sensitized rats. TDI-sensitized rats were treated with various doses of quercetin for 5 days before TDI provocation. Olopatadine hydrochloride (OH) was administered orally into TDI-sensitized rats once a day for 5 days at a single dose of 10 mg/kg before the provocation. The levels of SP in nasal lavage fluids obtained from sensitized rats 6 h after TDI nasal challenge were measured by ELISA. The data are expressed as the mean pg/ng IgA **±** standard errors of the means of five rats/group. NC: non-sensitized, non-treated control; EA: ethyl acetate

**Fig. 5:**
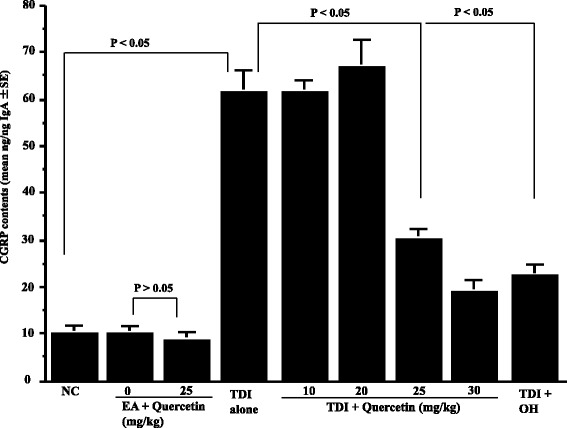
Influence of quercetin on the appearance of calcitonine gene-related peptide (CGRP) in nasal lavage fluids after toluene 2,4-diisocyanate (TDI) nasal instillation in sensitized rats. TDI-sensitized rats were treated with various doses of quercetin for 5 days before TDI provocation. Olopatadine hydrochloride (OH) was administered orally into TDI-sensitized rats once a day for 5 days at a single dose of 10 mg/kg before the provocation. The levels of CGRP in nasal lavage fluids obtained from sensitized rats 6 h after TDI nasal challenge were measured by ELISA. The data are expressed as the mean ng/ng IgA **±** standard errors of the means of five rats/group. NC: non-sensitized, non-treated control; EA: ethyl acetate

**Fig. 6:**
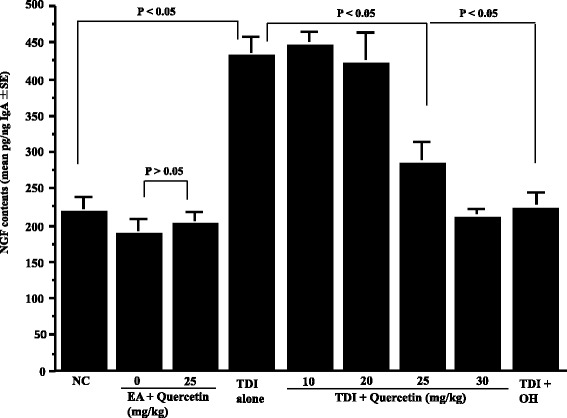
Influence of quercetin on the appearance of nerve growth factor (NGF) in nasal lavage fluids after toluene 2,4-diisocyanate (TDI) nasal instillation in sensitized rats. TDI-sensitized rats were treated with various doses of quercetin for 5 days before TDI provocation. Olopatadine hydrochloride (OH) was administered orally into TDI-sensitized rats once a day for 5 days at a single dose of 10 mg/kg before the provocation. The levels of NGF in nasal lavage fluids were measured by ELISA. The data are expressed as the mean pg/ng IgA **±** standard errors of the means of five rats/group. NC: non-sensitized, non-treated control; EA: ethyl acetate

The second set of experiments was undertaken to examine the duration of quercetin treatment on the appearance of neuropeptides in nasal lavage fluids obtained from sensitized rats after TDI nasal challenge. To do this, TDI-sensitized rats were orally administered quercetin at 25 mg/kg, which is the minimum concentration showing the suppressive effects on the development of nasal symptoms, for 2 to 7 consecutive days before the challenge. Nasal lavage fluids were obtained from rats 6 h after the challenge and SP levels were examined by ELISA. As shown in Fig. 3, oral administration of quercetin for more than 5 days significantly inhibited the increase in SP levels in nasal lavage fluids, which was increased by intranasal challenge with TDI. We then examine the dose response profile of quercetin on the appearance of SP in nasal lavage fluids. TDI-sensitized rats were orally administered various doses of quercetin for five consecutive days before the challenge. Nasal lavage fluids were obtained from rats 6 h after the challenge and SP levels were examined by ELISA. As shown in Fig. 4, oral administration of quercetin at more than 25 mg/kg caused significant decrease in SP levels in nasal lavage fluids, which was increased by TDI nasal challenge. It is also showed that oral administration of OH at 10 mg/kg for 5 days could inhibit increase in SP levels in nasal lavage fluids after TDI nasal challenge (Fig. 4). We then examined whether quercetin and OH could also suppress the appearance of neuropeptides, CGRP and NGF, in nasal lavage fluids after TDI nasal challenge as in the case of SP. TDI-sensitized rats were orally administered various doses of quercetin or 10 mg/kg OH for 5 days before the challenge. Nasal lavage fluids were obtained from rats 6 h after the challenge and the levels of CGRP and NGF were examined by ELISA. As shown in Fig. 5, oral administration of quercetin at more than 25 mg/kg into TDI-sensitized rats could decrease CGRP level in nasal lavage fluids, which was increased by TDI nasal challenge. It is also showed the suppressive effect of 10 mg/kg OH on CGRP appearance in nasal fluids after TDI nasal challenge (Fig. 5). Furthermore, oral administration of quercetin at more than 25 mg/kg into TDI-sensitized rats could also significantly suppress the increase in NGF contents in nasal lavage fluids after TDI nasal challenge as well as 10 mg/kg OH (Fig. 6).

## Discussion

AR is a global health problem that affects patients of all ages and characterized mainly by the three cardinal symptoms such as sneezing, nasal obstruction and watery rhinorrhea [1, 2]. These clinical symptoms are considered to be caused by the chemical mediators such as histamine, prostaglandins and leukotriens secreted from inflammatory cells especially mast cells [1, 2]. In addition to the development of clinical symptoms, these mediators stimulate sensory nerves to release several types of neuropeptides which in turn produce itching, nasal congestion and sneezing [3–6]. From these established concepts, histamine H_1_ receptor antagonists are recommended as a first choice of the agents for AR treatment as well as topical corticosteroids [2]. On the other hand, there is much evidence that oral administration of quercetin and its derivative, isoquercetin, into allergic patients could modify favorably the clinical conditions of the diseases and the therapeutic mode of quercetin may be owing, at least in part, to its suppressive effects on inflammatory cell (e.g. mast cells and eosinophils) activation [11, 12]. However, the influence of quercetin on the production of neuropeptides, which are responsible for the development of clinical symptoms of AR, is not fully understood. The present study, therefore, was undertaken to examine the influence of quercetin on neuropeptide production using TDI-sensitized rats.

The present results clearly showed that oral administration of quercetin at more than 25 mg/kg for 5 days into TDI-sensitized rats significantly inhibited the development of nasal allergy-like symptoms such as sneezing and nasal rubbing movements, which are induced by nasal antigenic challenge. The clinical nasal allergic reaction is divided into the early (or immediate)- and the late-phase response [2]. The early phase-allergic response is occurred within minutes of allergen exposure and characterized by sneezing, nasal itching and watery rhinorrhea, which are mainly caused by the chemical mediators secreted from mast cells and basophils [2]. It is also reported that the chemical mediators stimulate nasal sensory neurons to cause sneezing reflax via the central nervous system [3, 4, 6]. There is also a local effect, so called axonal reflax, in which sensory nerves release several types of neuropeptides such as SP and CGRP, leading to vasodilation and plasma exudation [3, 4, 6]. These responses originated in sensory nerve activation are called neurogenic inflammation [4]. Intranasal instillation of TDI into rats is well accepted to induce nasal neurogenic inflammation and used frequently for analysis the mechanisms of nasal neurogenic inflammation [17–19]. Together with these reports, the present results may be interpreted that oral administration of quercetin into TDI-sensitized rats attenuates the development of nasal neurogenic inflammation and results in inhibition of the development of nasal allergy-like symptoms induced by TDI nasal provocation in rats.

The second part of experiments was undertaken to evaluate the influence of quercetin on the development of nasal neurogenic inflammation by examining the contents of neuropeptides in nasal lavage fluids. The present data clearly showed that oral administration of quercetin at more than 25 mg/kg for 5 days could significantly inhibit the appearance of neuropeptides, SP, CGRP and NGF in response to TDI nasal challenge. SP and CGRP act synergistically and increase each other mast cell degranulation to produce chemical mediators responsible for development of the early-phase allergic response [6, 21]. These two neuropeptides also activate macrophages to produce pro-inflammatory cytokines, including interleukin (IL)-1β, IL-3 and TNF-α, which are functioned in additional stimulus to allergic inflammation [22, 23]. Furthermore, SP increases the ability of sensory nerves and nasal epithelial cells to produce NGF [24, 25], which contributes to amplifying the early-phase allergic response through the promotion of both survival and degranulation of mast cells [6, 25]. NGF also activates tyrosine kinase A (TrkA) receptor which in turn initiates signaling via the phosphatidylinositol 3 kinase/phosphatidylinositol phosphate 3 (PI3K/PIP3) pathway to increase expression and sensitivity of transient receptor potential vallinoid (TRPV1) receptor, which is responsible for the production and release of SP and CGRP from sensory neurons [6]. These reports may suggest that oral administration of quercetin at more than 25 mg/kg for 5 days into TDI-sensitized rats inhibit neuropeptide productions from sensory neurons and results in attenuation of the appearance of nasal allergy-like symptoms induced by TDI nasal provocation.

Although the present results strongly suggest that quercetin attenuates nasal allergy-like symptoms through the suppression of neuropeptide release after antigenic challenge, the precise mechanisms by which quercetin inhibits neuropeptide production from sensory nerves after antigenic challenge. It is reported that the activation of the extracellular signal-regulated kinase 1/2 (ERK1/2) pathway is essential for the production of both SP and CGRP in dorsal root ganglions and peripheral nerve fibers [26]. The activation of ERK1/2 and nuclear factor-κB (NF-κB) pathway is also reported to enhance SP production and is responsible for the development of neurogenic inflammation [26, 27]. Furthermore, NGF production in keratinocytes requires the activation of mitogen-activated protein kinase (MAPK), especially ERK1/2, and phosphorylation of NF-κB after stimulation with SP, CGRP and other inflammatory mediators [28]. Neuropeptides synthetized in the neuron cell body are stored in vesicles and the vesicles are transported to the release sites such as the axon terminals [29]. When an action potential reaches the axon terminals, Ca^2+^ is influx into the axon terminals through Ca^2+^ channels and increases the intracellular Ca^2+^ concentrations. And then neuropeptides are released by exocytosis to the extracellular space [28, 29]. It is reported that Ca^2+^ is an essential molecule for activation of both MAPK, including ERK1/2 and NF-κB after stimulation of cells with inflammatory molecules such as cytokines and neuropeptides [30]. Quercetin is reported to increase in intracellular Ca^2+^ contents after compound 48/80 stimulation in human mast cell line in vitro [31]. From these reports, there is possibility that oral administration of quercetin into TDI-sensitized rats inhibits changes in Ca^2+^ contents in the axon terminals after TDI nasal challenge and results in suppression of neuropeptide appearance in nasal lavage fluids.

Allergic diseases such as AR, asthma and atopic dermatitis (AD) are characterized by intense infiltration and activation of Th2 T-cells, mast cells and eosinophils into the inflamed tissues. Th2 T-cells are believed to orchestrate the development and maintenance of allergic inflammatory responses through the secretion of inflammatory cytokines such as IL-3, IL-5 and thymus and activation-regulated chemokine (TARC), which are responsible for promotion of migration and activation of inflammatory cells, including mast cells and eosinophils [1]. It is reported that quercetin at 5.0 μM significantly suppressed TARC production from human keratinocytes, HaCat cell after TNF-α stimulation in vitro [32]. Oral administration of 2.5 mM quercetin three times a week for 3 weeks into AD experimental model mouse, Nc/Nga is also reported to decrease serum TARC levels along with attenuation of clinical symptoms [32]. Furthermore, there is evidence that quercetin at 5.0 μM significantly suppresses eosinophil degranulation and the ability of eosinophil to produce chemokines such as eotaxin, RANTES and MIP-1β, which are implicated in development and persistence of eosinophilic inflammatory responses [1], after immunological and no-immunological stimulations in vitro [11, 12, 33–35]. In regard to mast cells functions, quercetin inhibits the release of histamine and several types of inflammatory cytokines responsible for the development of allergic responses [8, 35–37]. Together with these reports and the present results, it is strongly suggest that quercetin modulates the activation of inflammatory cells and neuropeptide productions and results in improvement of clinical conditions of allergic diseases, especially AR.

## Conclusion

The present results clearly showed the attenuating effects of quercetin on nasal allergy-like symptoms induced by neurogenic inflammation through the suppression of neuropeptide production in vivo. Thus, quercetin will be a useful supplement for the management and treatment of AR.

## Abbreviations

AD: atopic dermatitis
AR: allergic rhinitis
CGRP: calcitonin gene-related peptide
ERK1/2: extracellular signal-regulated kinase 1/2
IL: interleukin
MAPK: mitogen-activated protein kinase
NF-κB: nuclear factor-κB
NGF: nerve growth factor
OH: olopatadine hydrochloride
SD: Sprague-Dawley
SP: Substance P
TARC: thymus and activation-regulated chemokine
TDI: toluene 2,4-diisocyanate
